# Molecular epidemiology of *Plasmodium vivax *anti-folate resistance in India

**DOI:** 10.1186/1475-2875-10-102

**Published:** 2011-04-24

**Authors:** Surendra K Prajapati, Hema Joshi, Vas Dev, Virendra K Dua

**Affiliations:** 1 Genetics and Molecular Biology Laboratory, National Institute of Malaria Research (NIMR), Sector-8, Dwarka, New Delhi 110077, India; 2 NIMR field station, Assam, India

## Abstract

**Background:**

Sulphadoxine and pyrimethamine are anti-folate drugs that show synergistic anti-malarial effect. Point mutations in *dihydrofolate reductase *(*dhfr*) and *dihydropteorate synthatase *(*dhps*) cause anti-folate drug resistance phenotype in human malaria parasites. This study presents pattern of point mutations in *dhfr/dhps *genes among Indian sub-continent.

**Methods:**

Microscopically diagnosed one hundred *Plasmodium vivax *field isolates were collected from five widely separated geographical regions of India. *Dhfr *and *dhps *genes were PCR amplified and sequenced. Previously published mutations data were collected and analyzed using Chi square test to identify geographical cluster of mutant/wild type genotypes.

**Results:**

Sequence analysis revealed single (S58R), double (S58R/S117N) and quadruple (F57L/S58R/T61M/S117T/) point mutations at *dhfr *and single (A383G) to double (A383G/A553G) mutations at *dhps *in *P. vivax *field isolates. In addition, three new mutations were also observed at *dhfr*. Both, *dhfr *and *dhps *genes revealed tandem repeat variations in field isolates. *Dhps *revealed very low mutation frequency (14.0%) compared to *dhfr *(50.70%). Comparative analysis revealed a progressive increase in frequency of quadruple mutant *dhfr *genotype (p < 0.001) within five years in north-eastern state (Kamrup, Assam). Frequency of *dhfr *genotypes revealed three distinct geographical clusters of wild (northern India), double mutant (southern India), and quadruple mutant (north-eastern and island regions of India) on the Indian sub-continent.

**Conclusion:**

Study suggests that SP may be susceptible to *P. vivax *in India, except Andaman and north-eastern state. The distinction of geographical regions with sensitive and resistant parasite phenotypes would be highly useful for designing and administering national anti-malarial drug policy.

## Background

Malaria is a life-threatening ancient parasitic disease and causes 250-500 million clinical episodes and nearly one million deaths annually [1]. Among the five human malaria species, *Plasmodium falciparum *is the most severe form, causing malignant malaria globally, while *Plasmodium vivax *is the most widespread species outside Africa, causing huge morbidity and can be severe and fatal [2-7].

The worldwide spread of chloroquine (CQ) resistant strains of *P. falciparum *has led to use of sulphadoxine-pyrimethamine (SP) as the first-line anti-malarial drug in Southeast Asian countries. Sulphadoxine and pyrimethamine sequentially inhibits dihydropteroate synthase (DHPS) and dihydrofolate reductase (DHFR) enzymes respectively in the folate biosynthesis pathway resulting synergistic anti-malarial effect [8]. Parasite has overcome the effect of SP by evolving point mutations in the respective genes encoding enzymes involved in the folate biosynthesis pathway. The mutated DHPS and DHFR enzymes have reduced binding affinity with SP drug and thus parasite survives in the presence of drug [9,10].

In India, resistance to CQ was reported for the first time in 1973 in *P. falciparum *from north-eastern states [11] and later it spread throughout the country [12]. To overcome CQ resistance problem, in 1982, SP was employed as first-line anti-malarial therapy for treatment of falciparum malaria in areas with >25% CQ resistant level, complicated malaria case, and higher malaria endemicity [12]. Currently, according to the national anti-malarial drug policy, artimisinin-based combination therapy (ACT) (Artesunate+SP) is being used in most of the malaria endemic regions [12].

Reduced susceptibility to CQ in *P. vivax *was for the first time observed in 1989 in Australian soldiers returned from Papua New Guinea [13]. Later, several cases from Papua New Guinea, Indonesia, New Guinea, Brazil and India were documented [14-19]. Recent studies from *P. vivax ex-vivo *maturation experiment showed reduction in the susceptibility to CQ in Southeast Asian counties [20,21]. This information indicates gradual increase in CQ resistance cases of *P. vivax*.

India contributes more than 78% of total malaria cases of Southeast Asia and *P. vivax *accounts for more than 50% of annual malaria cases [22]. Point mutations in *P. vivax dhfr *had been documented from different parts of India [23-25], however mutation data of *dhps *is yet to identified in order to understand the molecular epidemiology of anti-folate resistance. Therefore, identifying information about presence of anti-folate drug resistance related point mutations (*dhfr/dhps*) in *P. vivax from *Indian sub continent would be highly helpful to understand the global pattern of anti-folate drug resistance. This study aims to identify point mutations in *dhfr/dhps *and the spatio temporal pattern of anti-folate drug resistance in Indian sub-continent.

## Methods

### Study sites and sample collection

Blood samples were collected from five widely separated geographical regions of the Indian subcontinent namely Delhi (2005); Chennai, Tamil Nadu (2005); Kamrup, Assam (2007); Nadiad, Gujarat (2005) and Panna, Madhya Pradesh (2006). Details of epidemiological and geographical information about study sites are reported elsewhere [26]. Finger prick blood was spotted on autoclaved Whatman filter paper strips (Number 3) from the symptomatic patients in active case detection surveys as well as from patient attending clinics. A total of 100 microscopically-diagnosed *P. vivax *positive blood samples were spotted and dried blood spots were stored at 4.0°C. Only *P. vivax *infected samples were included in this study. This study was approved by the ethics committee of the National Institute of Malaria Research, New Delhi. All blood spots were collected only after obtaining consent of the patients.

### DNA extraction, PCR, and DNA sequencing

Genomic DNA was extracted from blood spots using QIAamp mini DNA kit (Qiagen, Germany) according to manufacturer instructions. Genomic DNA was eluted in 120.0 μl triple sterile water and store in -20°C until use. One step modified PCR strategy was employed for amplification of *dhfr/dhps *using nested primers reported earlier [27,28]. The modified PCR conditions for *dhfr *was- initial denaturation at 95°C/5.0 minute, denaturation at 95°C/30 second, annealing at 64°C/30 second, and extension at 72°C/60 second for 40 cycle, and a final extension at 72°C/5.0 minute. The modified PCR amplification for *dhps *was same as *dhfr *except annealing step, which is 55°C/30 second. PCR products were purified with Exonuclease I/Shrip alkaline phosphates treatment according to manufacturer instruction (Fermentas, USA). Purified PCR products were outsourced to Macrogen Inc, Korea for DNA sequencing [29]. Each sample was sequenced with both forward and reverse primers. DNA sequences were edited and aligned (ClustalW method) with EditSeq and MegAlign module of DNA Lasergene software version 7.0 (Madison, USA). Samples that show novel point mutations were re-sequenced from another independent PCR product. All sequences have been submitted to the GenBank (EU149665-EU149764, EU145878-EU145947).

### Collection of *dhfr *mutations data and statistical analysis

*Dhfr *point mutations data from Panna, Madhya Pradesh (N = 30); Nadiad, Gujarat (N = 19); Mukherji Nagar, Delhi (N = 68); Cuttack, Orissa (N = 16); Mohan Nagar (N = 49) Aligarh (N = 38) and Mirzapur (N = 8), Uttar Pradesh; Chennai, Tamil Nadu (N = 67) Car Nicobar, Andaman Nicobar (N = 38); Panjim, Goa (N = 76); Kamrup, Assam (N = 24) and Navi Mumbai, Maharashtra (N = 24) were collected from the earlier studies [23-25,30]. On the basis of mutations in *dhfr *and geographical location, grouping between different geographical regions was done. Geographical regions were grouped according to the higher prevalence of wild type, double mutant, and quadruple mutant *dhfr *genotypes. Chi square test was done to check the significance of prevalence of *dhfr *mutant genotypes in geographical locations. Confidence interval at 95% was done to check the proportion *dhfr *genotypes in each group.

## Results

### Mutations analysis in *dhfr *and *dhps*

For *dhfr*, a total of 71 *P. vivax *isolates were successfully PCR amplified and sequenced. PCR amplified *dhfr *was 711 bp in length and covers complete coding region. All sequences were compared with wild type reference sequence (GenBank accession no. X98123) to detect point mutations in *dhfr*. Point mutations observed at *dhfr *were given in table 1. Approximately 50% (35/71) sequences at *dhfr *were wild type. DNA sequence alignment revealed point mutations at 57(F→L), 58(S→R), 61(T→M), 64(V→L), 117(S→T), and 173(I→F). Point mutations at codon 64 (V→L) and 173 (I→F) were first time observed in the Indian isolates. One isolate showed amino acid substitution (57F→L) by changing nucleotide TTG rather than TTA (TTC wild type). *Dhfr *sequence analysis revealed two synonymous mutations at codon 69Y (TAT→TAC) and 134 V (GTC→GTT). Synonymous mutation 134 V (GTC→GTT) is novel and observed only in the wild type *dhfr *genotype. The majority of the mutated codons were S58R (47.88%), S117N/T (46.47%), F57L (21.12%), and T61M (19.71%). Study isolates revealed single (S58R), double (S58R/S117N) and quadruple (F57L/S58R/T61M/S117T/) mutations.

**Table 1:** Distribution of *Plasmodium vivax dhfr/dhps *point mutations in field isolates

			DHFR										DHPS	
						
Regions	Sample size	Wild type	F57L	S58R	T61M	V64L	S117N/T	I173F	T69C	C134T	Sample size	Wild type	A383G	A553G
Nadiad	3	2		1			1				20	20		
Delhi	11	10				1			**2**		20	17	3	3
Panna	17	13		4			3		**8**	**2**	20	20		
Chennai	19	3	1	16	1		16		**1**		20	9	11	8
Kamrup	21	7	14	13	13		13	1			20	20		
Total	71	35	15	34	14	1	33	1	**11**	**2**	100	86	14	11

Boldface: synonymous substitution,

Values indicate number of isolates.

For *dhps*, a total of 100 *P. vivax *isolates were successfully PCR amplified (950 bp) which covers hotspot region for drug resistance related point mutations (codon 350 to 652). All sequences were compared with wild type reference sequence (GenBank accession no. AY186730) to detect point mutations in *dhps*. Sequence analysis revealed point mutation at codon A383G and A583G (Table 1). Majority of the sequences were wild type (86%). Only single (A383G) and double (A383G/A583G) point mutations were observed at *dhps *in the study isolates (Table 1).

### Geographical distribution of *dhfr *and *dhps *genotypes

Four distinct genotypes of *dhfr *were observed and these are wild type, single mutant, double mutant and quadruple mutant. In total, among mutant genotypes, frequency of double mutant (28.16%) and quadruple mutant (19.71%) was higher compared with single mutant (2.81%). Isolates of Panna, Delhi, and Nadiad were dominated by wild type genotype (Table 2). Frequency of mutant *dhfr *genotypes was highest in isolates of Chennai (84.24%) and Kamrup (66.67%) regions. In contrast, isolates from Delhi (9.0%), Panna (23.52%), and Nadiad (33.34%) showed relatively low proportion of mutant genotypes. Geographical distribution of various genotypes observed at *dhfr *is given in Table 2. Double mutant genotype was observed to be predominated in Chennai isolates (78.94%) whereas quadruple mutant genotype was dominated in Kamrup region (61.90%). The silent point mutation T69C was observed in wild type *dhfr *sequences only. The point mutation T69C was found at a high frequency in isolates of Panna (47.05%) but in lesser extent in Delhi (18.18%) and Chennai (5.26%) isolates. Another silent point mutation C134T was limited to isolates of Panna (11.76%).

**Table 2:** Region wise distribution of *Plasmodium vivax dhfr *and *dhps *genotypes

		DHFR (%)					DHPS (%)		
				
Region	Sample size	WT	SM	DM	QM	Sample size	WT	SM	DM
Nadiad	3	66.67		33.33		20	100		
Delhi	11	90.9	9.1			20	85		15
Panna	17	76.47	5.88	17.65		20	100		
Chennai	19	15.79		78.95	5.26	20	45	15	40
Kamrup	21	33.34		4.76	61.9	20	100		
Total	71	49.29	2.81	28.16	19.71	100	86	3	11

Three distinct *dhps *genotypes were observed in the field isolates: wild type, single mutant and double mutant. The wild type genotype was observed in high frequency in field isolates of Delhi (85%) and Chennai (55%) whereas isolates from Panna, Nadiad, and Kamrup were exclusively wild type. The mutant genotypes (single and double) were only observed in isolates from Delhi and Chennai (Table 2).

### Tandem repeat variation, distribution, and association with mutations

**Figure 1:**
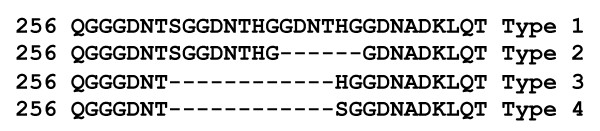
**Tandem repeat variation in *Plasmodium vivax *DHFR**.

At *dhfr*, four tandem repeat variants were observed and designated as Type 1-4, on the basis of six amino acid deletion/insertion (Figure 1). In total, tandem repeat Type 2 (84.50%) was the major repeat polymorph observed in field isolates (Table 3). Type 2 tandem repeat was observed in high frequency in all study sites except for Delhi where Type 1 predominates. Distribution of tandem repeat variants in different study sites is given in Table 3. Tandem repeat variant was monomorphic (Type-2) in field isolates of Nadiad, Chennai and Kamrup, whereas it was polymorphic in field isolates of Delhi and Panna. This suggests that monomorphic nature of tandem repeat was only observed in a region where mutant *dhfr *genotypes predominate. In study isolates, all mutant *dhfr *genotypes were observed in isolates having Tandem repeat Type-2.

**Table 3:** Distribution of tandem repeat variants at DHFR and DHPS in field isolates

DHFR	Regions (%)					Total N (%)
		
Repeat Type	Delhi	Nadiad	Panna	Chennai	Kamrup	
Type 1	54.54	0	11.76	0	0	8 (11.26)
Type 2	36.34	100	76.47	100	100	60 (84.5)
Type 3	9.09	0	0	0	0	1 (1.4)
Type 4	0	0	11.76	0	0	2 (2.81)
Total (N)	11	3	17	19	21	71
DHPS						
Type A	10	10	0	5	21.05	9.27
Type B	90	85	83.34	90	26.31	75.25
Type C	0	0	5.55	5	0	
Type D	0	5	5.55	0	15.79	5.15
Type E	0	0	0	0	10.52	2.06
Type F	0	0	0	0	10.52	2.06
Type G	0	0	5.55	0	10.52	3.09
Type H	0	0	0	0	5.26	1.03
Total (N)	20	20	18*	20	19*	97

*: DNA sequences of tandem repeat region was not good in some isolates and therefore not included in analysis of tandem repeats

**Figure 2:**
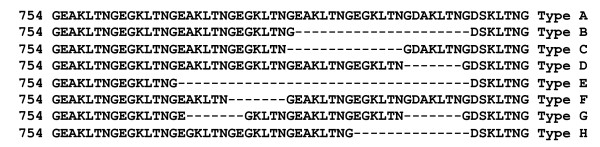
**Tandem repeat variation in *Plasmodium vivax *DHPS**.

At *dhps*, eight tandem repeats variants were observed and designated as Type A-H on the basis of deletion/insertion of seven amino acid repeat whereas (Figure 2). Tandem repeat Type-B was the major variant in the field isolates (75.25%) and predominate in all study sites (Table 3). Distribution of tandem repeat variants in different study sites is given in Table 3. Seven tandem repeat variants were found in field isolates of Kamrup, three in Panna, Chennai, and Nadiad and only two in Delhi. Point mutations were only observed in field isolates having tandem repeat Type B.

### Progressive increase in frequency of *dhfr *quadruple mutant

In Kamrup region, *dhfr *point mutation data from 2002-2003 revealed higher frequency of double mutant (54.16%) and low prevalence of wild (29.16%) and quadruple mutant (16.67%) genotypes [23]. Present study conducted in 2006-2007 revealed quite surprising result with respect to the data of *dhfr *point mutations studied in 2002-03. In the present study a high prevalence of the quadruple mutant genotype (61.90%) and an almost absence of the double mutant genotype (4.16%) were observed. However, the lower prevalence of wild type remains the same. The interesting finding was the drastic increase in the frequency of quadruple mutant *dhfr *genotype in Kamrup region (p = <0.0001) within a five year (2002 to 2007) span. This suggests that there is progressive increase in the frequency of quadruple mutant *dhfr *genotype.

### Geographical clustering of *dhfr *genotypes among Indian continent

**Figure 3:**
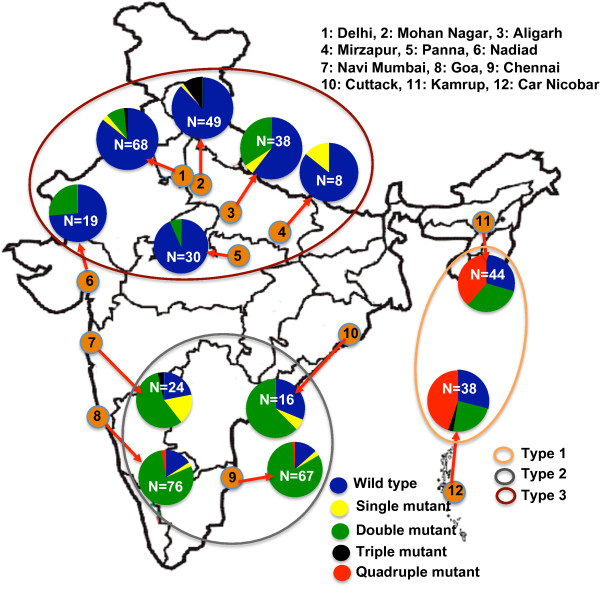
**Frequencies of *Plasmodium vivax dhfr *genotypes and their geographical clustering among Indian subcontinent**.

*Dhfr *point mutations data of earlier published work (n = 477) revealed single to quadruple mutant genotypes with their varied prevalence in different geographical regions of India (Figure 3). Comprehensive analysis of *dhfr *point mutations revealed three distinct pattern which are- 1) high prevalence of wild type, 2) high prevalence of double mutant type, and 3) high prevalence of quadruple mutant type. On the basis of above categorization, study sites were grouped together to see any trend of significance between geographical location and prevalence of *dhfr *genotypes. Three distinct geographical clusters were appeared at significant level (p < 0.0001). These geographical clusters are- Type-1) covers geographical areas of North India and majority of isolates were wild type (81.64%, 95% CI = ± 4.9) and very low proportion of single and or double mutant. Type-2) covers geographical areas of South India and majority of isolates were double mutant type (80.54%, 95% CI = ± 6.22) and very low proportion of wild and single mutant types. Type-3) covers geographical areas of north-eastern state and Island region where quadruple mutant (42.68%, 95% CI = ± 10.72) predominate over wild (29.26%) and double mutant type (28.04%). Details of geographical regions falling in specific cluster are given in figure 3. Comparison of Type-1 cluster with Type-2 or mutant versus wild types (p < 0.0001), and Type-2 cluster with Type-3 or double mutant versus quadruple mutant (p < 0.0001) strongly supports grouping or clustering of wild, double mutant, and quadruple mutant *dhfr *genotypes in three distinct geographical regions of the country.

## Discussion

Due to emergence and widespread of drug resistant strains of human *Plasmodium *species against chemotherapeutic intervention agents, clinical trial for monitoring drug efficacy over regular intervals as well as monitoring mutation data in relevant genes related with anti-malarial resistance are the crucial steps in deciding scope of a drug in field. This study uncovers 1) progressive increase in the frequency of quadruple mutant *dhfr *genotypes, 2) limited point mutations in *dhps *among Indian isolates, and 3) distinct pattern of anti-folate resistance in Indian sub-continent linked with geographical regions.

*Plasmodium vivax *is still susceptible to chloroquine in India [30,31] and anti-folate drugs are not used for treatment of vivax malaria, therefore, the appearance of point mutations in *dhfr/dhps *is surprising. This has been explained earlier because of the use of sulphadoxine-pyrimethamine to treat chloroquine-resistant *P. falciparum *is creating selection pressure in the *P. vivax *population. This has been reflected in the isolates of areas with sympatricity of *P. falciparum *and *P. vivax *[23,25], which presented a higher proportion of double and quadruple mutant *dhfr *genotypes than other regions. Cotrimoxazole (sulfamethoxazole and trimethoprim), an anti-microbial drug that target bacterial *dhfr/dhps*, is widely used in bacterial infection as prophylaxis, and has potential to select mutant *dhfr/dhps *of malaria parasites [32,33]. Cotrimoxazole is being used in India to treat bacterial infection in wide spectrum [34,35], which suggests that it could be a potential factor to select anti-folate point mutations in the Indian subcontinent. Other factors, i.e. presumptive treatment of malaria without diagnosis and use of S/P by private practitioners, cannot be ruled out.

The comprehensive analysis of point mutations in *dhfr *among Indian sub-continent revealed three distinct clusters/groups/types linked with geographical locations. North and south India revealed a prevalence of wild and double mutant *dhfr*, respectively, whereas north-eastern and island regions revealed a high prevalence of quadruple mutant *dhfr *genotype. Single to quintuple mutations have been reported in *dhfr *but only triple to quintuple mutant alleles are known to confer high level of resistance [36-38]. This strongly suggests that *P. vivax *isolates from northern and southern part of India could be sensitive to anti-folate drug, whereas isolates from north-eastern state and Island regions will be resistant to anti-folate drug.

A significant association between *dhfr *mutations and tandem repeat polymorphism was observed in studied isolates. The mutated *dhfr *genotype was exclusively associated with Type-2 tandem repeat and is strongly supported by previous study from India [23]. Further quadruple mutant *dhfr *alleles were exclusively associated with type 1 tandem repeat and are supported by earlier studies from India [23], Thailand [38] and Myanmar [39], however exception to this association was only showed by a single isolate from Myanmar [39]. These observations speculate *dhfr *allele with type 1 tandem repeat could be more prone for mutations and development of higher level of resistance conferring genotypes. Therefore, it could act as molecular marker to predict the risk of mutant/higher level resistance conferring mutant genotypes in any geographical area of Indian subcontinent.

Frequency of quadruple mutant *dhfr *genotype was progressively increased in Kamrup isolates from 2005 to 2007 [23]. The high proportion of quadruple mutant *dhfr *genotype in endemic area may be: 1) because of the characteristics of mutant parasites associated with higher gametocytogenesis [40-43] which favour transmission of mutant parasites compared to wild type *dhfr*, and 2) north-eastern states of India are highly endemic for malaria and international borders with neighbouring countries surround the region. The borders are very porous and illegal migrations of people across the borders are very common thus, importing drug resistant strains. The higher frequency of quadruple mutant *dhfr *genotypes in the isolates of Myanmar (71%) and Thailand (96%) [39,44], supports for inflow of quadruple mutant *dhfr *genotype in north-eastern states of India.

The development and spread of drug resistant parasite strains is a major obstacle to the malaria elimination programme. Therefore, it is essential to identify drug resistance areas/regions on the basis of point mutations in order to manage the national anti-malarial drug policy. In this regard, this study revealed a higher and increasing prevalence of quadruple mutant *dhfr *genotype in north-eastern and Island regions, therefore, additional caution may be taken for treatment of vivax malaria in these regions to stop the flourishing of quadruple mutant *dhfr *genotypes in remaining part of country. The study concluded that *P. vivax *isolates from northern and southern part of India could be susceptible to SP except for the isolates from Car Nicobar (Island) and Assam (north-eastern region). Geographical clustering of *dhfr *mutant genotypes would provide a rationale for a more appropriate national anti-malarial drug policy.

## Competing interests

The authors declare that they have no competing interests.

## Authors' contributions

SKP: Experiment design, experimental work, data analysis, manuscript writing

HJ: Conceptual design of work, data analysis, manuscript writing,

VD: Sample collection, parasite identification, and experimental design

VKD: Conceptual design of work, Overall supervision of work, and manuscript writing

All authors read and approved the final manuscript.
